# The Peridental Membrane

**Published:** 1889-05

**Authors:** H. W. Crydermand


### The Peridental Membrane.

BY H. W. CRYDERMAND, D.D.S.

Read before the Detroit Dental Society, January 14th, 1889.

This is the appellation of a vascular fibrous tissue which covers that portion of the teeth situated beneath the gums denominated roots. Its functions are to unite the roots to the alveolus; to act as a cushion and lining between making an immovable joint between tooth and its bony socket; another function is to receive and to transmit to nerve centres all shocks from blows, etc., and by it we determine the amount of pressure that can be exerted on any particular tooth without injury.

The space between root and alveolus increases as the apex of the root is reached and is called the "apical space." Its blood supply is obtained from a branch of the same artery that supplies the pulp, branching off just before it enters the foramen and the terminal filaments anastomosing with the arteries that supply the gums, and just at the rim of the alveolus there is a rich plexus formed by union with the arteries of periosteum and gums—the "gingival plexus."

Thus it is demonstrated that the teeth have three sources of nourishment through the pulp, through the peridental membrane in its two sources.

This membrane has also two sources of nerve supply corresponding closely with its blood supply, excepting that the chief one is through the alveolus from the gums. This is nicely illustrated by a case cited by Dr. Black (page 920 vol. 1st American System of Dentistry). A young lady had lost the pulp of a first bicuspid at a time when the foramen was widely open. A piece of cotton was accidentally pushed through into the apical space. It was found necessary to cut through the alveolar wall and every thing was removed from the space. The pulp was gone; the nerves entering membrane by way of apical space was gone, and yet upon recovery the sense of touch was normal, being transmitted by way of gums."

The diseases are classified differently by various authors, but the principal one is inflammation or periodontitis, and the number of its causes are according to Dr. Flagg seventeen, viz:

1. Want of occlusion; 2. Mal occlusion; 3. Tartar; 4. Looseness of teeth; 5. Induration of tooth tissue; 6. Cavities of decay impinging on cementum; 7. Mechanical irritation as by blows, etc.; 8. Dental manipulation as leaving legature on; 9. Excess of filling material; 10. Inflammation of pulp; 11. Extraction of pulp without hemorrhage; 12. External irritation by forcible removal of pulp—enlarged foramen; 13. Putrescent pulp; 14. Previous peridontitis; 15. Action of medicines locally; 16. Action of medicines systemically; 17. Action of virus—syphilis, fever, diphtheria.

Of all the causes here enumerated the one numbered 13, putrescent pulp, is the most frequent and to it we direct attention in the discussion. A patient presents himself with a throbbing pain in the jaw; examination of the teeth reveals one or more particularly sore; surrounding gums quite red and often hot; the offending tooth may or may not have a cavity of decay but we will assume it does, and our diagnosis would be peridontitis caused by dead pulp. Our knowledge, whether obtained from our own experience or from others, designates the way of relief, first by drilling into pulp chamber if closed, giving vent to gas generated by putrescence of pulp, and the pain produced by drilling may be lessened by tying a ligature around the tooth and allowing the patient to pull up or down, as the case may be, just enough to balance pressure exerted on the drill.

After debris from cavity has been removed and cavity cleansed it is the writer's opinion it is better to give absolute rest of parts for a few hours to allow the soreness to subside; then carefully and thoroughly remove pulp from roots, cleanse canal with tepid water, after with alcohol or glycerine, but if pain has not fully abated a few drops of the oil of cloves and sulphate of morphia may be placed into canals with a good effect. A plan pursued by many is to place carbolic acid in the canal, but the only good it can do is to disinfect the canals and harm may be done by forming a clot if hemorrhage occurred.

Now may be applied with good results, stimulants to the gums, over the tooth such as capsicum bag or plaster, or counter-irritants, as iodine, or sedatives as aconite. The systemic treatment would be derivative in some form, as either direct blood letting by scarifying the gums, or the use of some cathartic or diuretic, or by the use of leeches, which is greatly admired by many practitioners. Opium in some of its forms may be administered to induce sleep then by giving rest to the tooth and membrane when resolution occurs which may be in a few hours or many days. There remains but to fill the canals and cavity of decay with suitable filling material according to the operator's judgment or patient's ability to pay.

